# FCR (Fludarabine, Cyclophosphamide, Rituximab) regimen followed by ^90^yttrium ibritumomab tiuxetan consolidation for the treatment of relapsed grades 1 and 2 follicular lymphoma: a report of 9 cases

**DOI:** 10.1186/1756-9966-30-16

**Published:** 2011-02-08

**Authors:** Francesco Pisani, Carlo Ludovico Maini, Rosa Sciuto, Laura Dessanti, Mariella D'Andrea, Daniela Assisi, Maria Concetta Petti

**Affiliations:** 1 Department of Hematology Regina Elena National Cancer Institute, Via Elio Chianesi, 53 00128 Rome, Italy; 2 Department of Nuclear Medicine Regina Elena National Cancer Institute, Rome, Italy; 3 Department of Gastroenterology Regina Elena National Cancer Institute, Rome, Italy

## Abstract

**Background:**

This retrospective analysis is focused on the efficacy and safety of radioimmunotherapy (RIT) with Zevalin^® ^in nine patients with recurrent follicular lymphoma (FL) who were treated in a consolidation setting after having achieved complete remission or partial remission with FCR.

**Methods:**

The median age was 63 yrs (range 46-77), all patients were relapsed with histologically confirmed CD20-positive (grade 1 or 2) FL, at relapse they received FCR every 28 days: F (25 mg/m^2^x 3 days), C (1 gr/m^2 ^day 1) and R (375 mg/m^2 ^day 4) for 4 cycles. Who achieved at least a partial remission, with < 25% bone marrow involvement, was treated with ^90^Yttrium Ibritumomab Tiuxetan 11.1 or 14.8 MBq/Kg up to a maximum dose 1184 MBq, at 3 months after the completion of FCR. The patients underwent a further restaging at 12 weeks after ^90^Y-RIT with total body CT scan, FDG-PET/CT and bilateral bone marrow biopsy.

**Results:**

Nine patients have completed the treatment: FCR followed by ^90^Y-RIT (6 patients at 14.8 MBq/Kg, 3 patients at 11.1 MBq/Kg). After FCR 7 patients obtained CR and 2 PR; after ^90^Y-RIT two patients in PR converted to CR 12 weeks later. With median follow up of 34 months (range 13-50) the current analysis has shown that overall survival (OS) is 89% at 2 years, 76% at 3 years and 61% at 4 years. The most common grade 3 or 4 adverse events were hematologic, one patient developed herpes zoster infection after 8 months following valacyclovir discontinuation; another patient developed fungal infection.

**Conclusions:**

Our experience indicate feasibility, tolerability and efficacy of FCR regimen followed by ^90^Y-RIT in patients relapsed with grades 1 and 2 FL with no unexpected toxicities. A longer follow up and a larger number of patients with relapsed grades 1 and 2 FL are required to determine the impact of this regimen on long-term duration of response and PFS.

## Background

Follicular lymphoma is the most common type of indolent non-hodgkin lymphoma (NHL) in Western countries and is typically characterized by recurrence of disease. There is usually a pattern of repeated remissions and relapses until patients become refractory to treatment. The duration of remissions becomes shorter with repeated induction attempts. Transformation to more aggressive NHL occurs in 15% to 50% of the patients at 5 years.After first relapse patients in otherwise good health are candidate for salvage chemotherapy: combination chemotherapy, immunotherapy, and for some patients with good performance status and responsive disease, myeloablative therapy with stem-cell rescue. A number of cytotoxic agents in combination are active in this patient population and FCR regimen has provided encouraging results as initial or salvage therapy in patients with CLL or indolent NHL [1,2]. Radioimmunotherapy is also an excellent modality in the treatment of NHL; the target antigen, radionuclide emission properties, and chemical stability of radioimmunoconjugates are important factors that contribute to the effectiveness of RIT.^90 ^Yttrium can deliver a high beta energy to tumor (2-3 MeV) and ^90 ^Yttrium Ibritumomab Tiuxetan ( ^90 ^Y -RIT ) - Zevalin^® ^- consists of the anti-CD20 monoclonal antibody ibritumomab (an IgG1k antibody which is the murine parent immunoglobulin to rituximab) covalently bound to the chelating agent tiuxetan and radiolabeled with ^90 ^Yttrium.

Furthermore recently FIT study has shown that consolidation of first remission with ^90 ^Yttrium in advance-stage follicular lymphoma is highly effective with no unexpected toxicities, prolonging progression free survival (PFS) by 2 years [3,4]. Then consolidation with ^90 ^Yttrium after first line induction therapy, may allow more patients, with disseminated disease at diagnosis, to benefit from radioimmunotherapy and may present an attractive treatment option, particulary in older patients (age ≥ 60 years) who represent rougly 50% of patients with newly diagnosed indolent NHL.

^90 ^Y-RIT also has been reported to be effective in patients with relapsed or refractory FL [5-7]. In this article we describe our experience with ^90 ^Y -RIT consolidation in nine patients relapsed with grade 1 and 2 FL patients, responding to FCR.

## Methods

### Patients

The patients who were included in the current retrospective analysis had CD20+ histologically confirmed relapsed grade 1 or 2 follicular lymphoma, all patients provided informed consent according to institutional guidelines. Patients had received at least one prior treatment, were age ≥ 18 years, with WHO performance status of 0 to 2, had achieved at least PR at the completion of FCR; the last chemotherapy with or without rituximab was administered at least three months before start of FCR; no patient under maintenance therapy with rituximab was considered. Patients had less than 25% bone marrow involvement by lymphoma on biopsy before start of RIT; an absolute neutrophil count ≥ 1.5 × 10^9 ^L; hemoglobin levels ≥ 9 gr/dl and a platelet count ≥ 100 × 10^9 ^L. Patients with central nervous system (CNS) involvement, positive HIV were excluded from the analysis.

### Treatment

**Figure 1:**
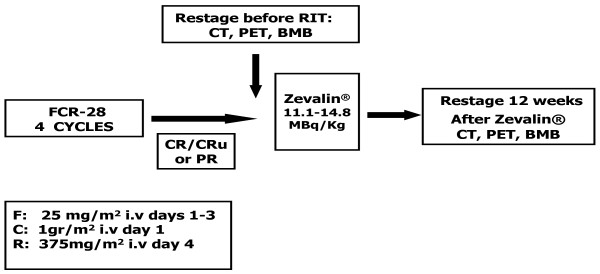
**Treatment schema**.

Patients at relapse had received 4 cycles of FCR: fludarabine at a dose of 25 mg/m^2 ^i.v. on days 1 to 3; cyclophosphamide at a dose of 1 gr/m^2 ^i.v. on day 1 and rituximab at a dose of 375 mg/m^2 ^was given on day 4 of each cycle every 28 days. Patients were restaged with CT scan, FDG PET/CT and bone marrow biopsies after the last course of FCR: who had achieved at least a partial remission, with < 25% bone marrow involvement, received 12 weeks since the last course of FCR two infusions of rituximab 250 mg/m^2 ^one week apart, with the first infusion administered alone and the second infusion followed immediately by ^90 ^Y-RIT 14.8 MBq/Kg - 11 MBq/Kg, if the platelet number was between 100 × 10^9^/L and 149 × 10^9^/L, not to exceed a total of 1.184 MBq administered as a slow i.v. push over 10 minutes (Figure 1).

### Assessments

All patients included in the analysis were restaged with CT scan, FDG-PET and bilateral bone marrow biopy at 4-5 weeks after the last cycle of FCR and 12 weeks after ^90 ^Y-RIT. No real-time quantitative PCR (RQ-PCR) evaluation of pheripheral or marrow blood samples for bcl-2 t(14;18) translocation was performed at baseline and thereafter. Safety was assessed by adverse events (AEs), with toxicity grading based on the National Cancer Institute Common Toxicity Criteria (version 2), clinical laboratory evaluations, and physical examinations. OS was calculated from the date of FCR treatment to the date of death from any cause; OS was analyzed by using the Kaplan-Meier method.

## Results

### Patients characteristics

In this retrospective analysis, from August 2005 to July 2010, 9 patients had received FCR 4 cycles followed by ^90 ^Y-RIT (6 patients at 14.8 MBq/Kg, 3 patients at 11.1 MBq/Kg). Baseline characteristics are presented in (Table 1). The median age was 63 years (range 46-77). All patients were relapsed patients: 2 patients received a prior therapy, 5 patients received 2 prior treatments and 2 patients had received 3 regimens. Seven patients were previously treated with rituximab plus chemotherapy, two patients had no previous rituximab treatment history, one patient received also high-dose therapy followed by autologous stem cell transplantation (Table 2)

**Table 1:** Patient characteristics

	Number of patients = 9	
**Male/Female**	**3/6**	

**Median Age (Range)**	**63 (46-77) years**	
**Disease stage**	**at diagnosis**	**at start of FCR**
**I**	**1**	**0**
**II**	**1**	**5**
**III**	**1**	**3**
**IV**	**6**	**1**
**Bone marrow involvement**		
**0%**	**7**	
**10% to ≤ 25%**	**2**	
**Extranodal involvement**	**1 (liver)**	

**FLIPI**		
**Low**	**1**	
**Low-intermediate**	**6**	
**Intermediate-high**	**2**	
**Bulky disease**	**1**	
**B-symptoms**	**0**	

**Prior therapy including rituximab**		
**No**	**2**	
**Yes**	**7**	
**Number of previous regimens**		
**1**	**2**	
**2**	**5**	
**>2**	**2**	

**Table 2:** Clinical characteristics

Patients No	Sex/Age (y)	Previous treatment	Response to FCR	Response to RIT	Follow up (mo) since RIT
**1**	**F/68**	**CHOP/R, radiotherapy**	**CR**	**CR**	**50 alive in CR**
**2**	**F/66**	**Radiotherapy, CHOP/R**	**CR**	**CR**	**34 alive in CR**
**3**	**F/57**	**CHOP/R**	**PR**	**CR**	**44 alive in CR**
**4**	**F/67**	**CHOP/R, radiotherapy**	**CR**	**CR**	**13 dead in CR**
**5**	**M/46**	**CHOP/like, ASCT, IFN maintenance for 24 months**	**PR**	**CR**	**28 alive in CR**
**6**	**F/61**	**MACOPB/R**	**CR**	**CR**	**39 alive in CR**
**7**	**M/69**	**CHOP,FM/R, Cy Dex/R**	**CR**	**CR**	**30 dead in CR**
**8**	**M/57**	**Chlorambucil, MACOPB/R**	**CR**	**CR**	**(t-MDS) 32 dead in CR**
**9**	**F/77**	**Chlorambucil, radiotherapy**	**CR**	**CR**	**44 alive in CR**

*CHOP*: cyclophosfamide, doxorubicin, vincristine, prednisone; *R*: Rituximab; *MACOPB*: Methotrexate, Doxorubicin, cyclophoshamide, vincristine, prednisone, bleomycin,; *ASCT*: autologous stem cell transplantation; *IFN*: alpha interferon, *FM*: fludarabine, mitoxantrone; *Cy Dex*: cyclophosphamide, dexamethasone; t-MDS: treatment-related myelodysplastic syndrome

### Efficacy and safety

After 4 cycles FCR seven patients obtained CR and 2 PR, two patients in PR converted to CR after RIT. With a median observation period of 34 months (range 13- 50) the OS is 89% at 2 years, 76% at 3 years and 61% at 4 years. Grade 3 or 4 neutropenia occurred in 8/9 patients treated with FCR and in 9/9 patients assessable after ^90 ^Y-RIT. Subsequently to radioimmunotherapy the median neutrophil nadir was 0.8 × 10^9^/L (range 0.1-0.9 × 10^9^/L) at week 5, the median platelet count nadir was 49 × 10^9^/L (range 17-80 × 10^9^/L) at week 5. The median duration nadir for both neutrophils or platelets was 14 days. One patient developed herpes zoster infection after 8 months following valacyclovir discontinuation; another patient developed fungal infection. Both infections disappeared after specific treatment. After a median observation period of 34 months one patient developed t-MDS (treatment-related myelodysplastic syndrome) at 26 months after ^90 ^Y-RIT. This patient before FCR and consolidation with RIT had received three previous regimens: at diagnosis 6 courses of CHOP, at first relapse, 3 years later, four courses of FM/R (fludarabine, mitoxantrone plus rituximab) and after one year, at the second relapse, he received cyclophosphamide plus dexamethasone and rituximab, remaining in CR for 48 months. He died at 73 years of age for sepsis during support therapy for t-MDS. Other two patients have died: one for acute renal failure and one for ictus cerebri.

## Discussion

In follicular lymphoma retreatment typically yields progressively less satisfactory responses than the prior treatment, eventually leading to refractory disease, and the question remains regarding whether the survival of patients with FL is improving with new treatment regimens.

In the current retrospective analysis, nine patients with relapsed grade 1 and 2 FL, responding to FCR regimen and consolidated with ^90 ^Y-RIT obtained a significant high rate of response with 100% of CR and acceptable toxicity. After a median observation period of 34 months 6/9 patients were alive in CR and 7/9 were already treated with at least two prior regimens. Two patients converted PR to CR after consolidation with ^90 ^Y-RIT. This conversion was already shown in published phase III study (FIT-study) in first-line FL [3,4], and in previous phase II studies of consolidation with the radioimmunotherapy agent ^131 ^I-tositumomab after first-line induction [8,9],

confirming the ability of ^90 ^Y-RIT to improve responses also in patients who are pretreated with rituximab based combination therapy [3]; even if in our two patients there is no proof that this conversion was due to RIT and not to a late response to FCR. In the FIT study close to 17% of the patients in the control arm, converted from PR to CR during watchful waiting [3], but it is to be considered that our two patients had higher risk of resistance being already pretreated.

In our analysis the OS at 2 years was 89%, at 3 years 76% and at 4 years 61%. In another study conducted on patients with recurrent FL, treated with FCR, a 75% OS rate at 4 years and a 61% PFS rate at 4 years were registered, but in that study only 7% of patients had been treated previously with rituximab and furthermore no patients had received combination treatment with chemotherapy plus rituximab [10]. Regarding AEs there was a high incidence of neutropenia and thrombocytopenia but hematologic toxicities grade 3 or 4 did not require transfusion but growth factor support was utilized in the majority of patients during FCR treatment, and in all of them after ^90 ^Y-RIT. Despite the high incidence of grade 3 or 4 neutropenia there were no patients requiring hospitalization for infection. We registered a case of herpes zoster infection after 8 months following valacyclovir discontinuation that disappeared after retreatment, and a case of fungal infection by *conidiobolus*, developed 10 months after ^90 ^Y-RIT and disappeared with itraconazole treatment. Other previous studies have already shown the low percentage of patients requiring hospitalization for infections [3,5] and a favorable safety profile [11,12]. A case of t-MDS with complex karyotype was diagnosed 26 months after ^90 ^Y-RIT consolidation: this patient received 3 previous regimens before FCR plus ^90 ^Y-RIT, as already mentioned he died for sepsis. This patient had been previously treated with topoisomerase II inhibitors, alkylating agents and purine nucleoside analogs. Czuczman et al. reported an incidence of t-MDS and t-AML (treatment-related acute myeloid leukemia) after ^90 ^Y-RIT of 0.3% per year after the diagnosis of NHL and 0.7% per year after treatment. Most patients with t-MDS or t-AML had multiple cytogenetic aberrations, commonly on chromosomes 5 and 7, suggesting an association with previous exposure to chemotherapy. In Czuczman study these malignancies were diagnosed at a median of 5.6 years (range 1.4 to 13.9) after the diagnosis of NHL and 1.9 years (range 0.4 to 6.3) after radioimmunotherapy [13]; the conclusion of this study was that the annualized incidences of t-MDS and t-AML were consistent with that expected in patients with NHL who have had extensive previous chemotherapy and do not appeared to be increased after ^90 ^Y-RIT. Cytogenetic testing before treatment with RIT may identify existing chromosomal abnormalities in previously treated patients, particularly those who have been treated with alkylating agents and purine analogs and would be at higher risk to develop t-MDS or t-AML.

In our series the other two death were not in relation of progressive disease and all three deceased patients obtained CR before ^90 ^Y-RIT and died still in CR. Additional follow up is required to determine potential long-term AEs with ^90 ^Y-RIT consolidation. In our patients, the response to ^90 ^Y-RIT was assessed by CT, bone marrow biopsies and also with FDG-PET, this imaging procedure is useful to evaluate disease extension before treatment and response to RIT in FL. A recent study has shown that the post-RIT PET result is an independent predictive factor of PFS [14].

## Conclusions

This retrospective analysis of nine relapsed grades 1 or 2 FL patients with median age 63 years, heavily pretreated, demonstrates that FCR followed by ^90 ^Y-RIT was feasible, safe and yielded high overall and complete response rates in patients with recurrent FL. Hematologic toxicity occurring with FCR or with RIT were clinically controllable and acceptable in a population composed mainly of patients with a history of prior treatment using rituximab plus chemotherapy. A longer follow up and a larger number of patients with relapsed grades 1 and 2 FL are required to determine the impact of this regimen on long-term duration of response and PFS, but this preliminary results suggest that this regimen could be an option to be used for the treatment in this setting of patients, specially at age of 60-75 and earlier in first relapse; further studies will help to clarify the best strategy for incorporating RIT into the treatment algorithm of these patients.

## Abbreviations

FCR: fludarabine cyclophosphamide rituximab; FL: follicular lymphoma; NHL: non hodgkin lymphoma; RIT: radioimmunotherapy; MeV: megaelectronvolt; MBq: megabecquerel; OS: overall survival; PFS: progression free survival; t-MDS: treatment related myelodisplastic syndrome.

## Competing interests

The authors declare that they have no competing interests.

## Authors' contributions

Conception and design: FP, wrote the paper

Provision of study materials or patients: FP, MCP, CLM, RS, LD, MD, DA

All authors have read and approved the final manuscript.
